# Microbiology, ecology, and application of the nitrite-dependent anaerobic methane oxidation process

**DOI:** 10.3389/fmicb.2012.00269

**Published:** 2012-07-27

**Authors:** Li-Dong Shen, Zhan-Fei He, Qun Zhu, Dong-Qing Chen, Li-Ping Lou, Xiang-Yang Xu, Ping Zheng, Bao-Lan Hu

**Affiliations:** ^1^ Department of Environmental Engineering, Zhejiang UniversityHangzhou, China; ^2^ Institute of Life Science, Zhejiang Chinese Medical UniversityHangzhou, China

**Keywords:** “NC10” phylum, unique ultrastructure, intra-aerobic pathway, greenhouse gas control, nitrogen removal

## Abstract

Nitrite-dependent anaerobic methane oxidation (n-damo), which couples the anaerobic oxidation of methane to denitrification, is a recently discovered process mediated by “*Candidatus* Methylomirabilis oxyfera.” *M. oxyfera* is affiliated with the “NC10” phylum, a phylum having no members in pure culture. Based on the isotopic labeling experiments, it is hypothesized that *M. oxyfera* has an unusual intra-aerobic pathway for the production of oxygen via the dismutation of nitric oxide into dinitrogen gas and oxygen. In addition, the bacterial species has a unique ultrastructure that is distinct from that of other previously described microorganisms. *M. oxyfera*-like sequences have been recovered from different natural habitats, suggesting that the n-damo process potentially contributes to global carbon and nitrogen cycles. The n-damo process is a process that can reduce the greenhouse effect, as methane is more effective in heat-trapping than carbon dioxide. The n-damo process, which uses methane instead of organic matter to drive denitrification, is also an economical nitrogen removal process because methane is a relatively inexpensive electron donor. This mini-review summarizes the peculiar microbiology of *M. oxyfera* and discusses the potential ecological importance and engineering application of the n-damo process.

## INTRODUCTION

Methane (CH_4_) is an important greenhouse gas, which has, so far, contributed an estimated 20% to global warming (Knittel and Boetius, 2009). In the past, the oxidation of methane was believed to be restricted to oxic environments. This view gradually changed with the discovery of anaerobic methane oxidation (AMO) coupled to sulfate reduction in anoxic marine sediments and water columns (Martens and Berner, 1974; Reeburgh, 1976; Valentine and Reeburgh, 2000).

In 2006, a new AMO process, nitrite-dependent anaerobic methane oxidation (n-damo), which couples AMO to denitrification, was discovered in an enrichment culture (Raghoebarsing et al., 2006). Several peculiar properties of the n-damo process have been discovered that make AMO of particular interest to those interested in microbiology, ecology, and environmental engineering: (i) the discovery of a new species (“*Candidatus* Methylomirabilis oxyfera”) linking the carbon and nitrogen cycles (Ettwig et al., 2010; Wu et al., 2011a), (ii) the potential contribution of the n-damo process to reduction of global warming via oxidization of methane to carbon dioxide (CO_2_), and (iii) the potential application of n-damo for nitrogen removal from wastewater by using methane instead of organic matter as an electron donor to drive denitrification.

This mini-review summarizes the microbiology of the n-damo process, including the phylogenetic affiliations, physiological and ultrastructural properties of *M. oxyfera*, and the molecular mechanisms of its intra-aerobic metabolism. In addition, this mini-review discusses the potential ecological importance of the n-damo process in natural ecosystems and the application of the process for wastewater nitrogen removal.

## DISCOVERY AND SURVEY OF THE RESPONSIBLE BACTERIA

Due to thermodynamic considerations, it was believed that autotrophic microorganisms capable of using nitrate (nitrite) as an electron acceptor for methane oxidation could exist in nature because nitrate (nitrite) is a more favorable electron acceptor than most other relevant electron acceptors under anoxic conditions (Strous and Jetten, 2004).

In 2006, Raghoebarsing and colleagues first reported an n-damo enrichment culture from anoxic freshwater sediment (Raghoebarsing et al., 2006). In this culture, one bacterial phylotype belonging to the candidate division “NC10” constituted 80% of the population. This division had been formed only by environmental sequences (Rappé, 2003). A smaller fraction of the population (up to 10%) consisted of *Archaea* that were distantly related to the AMO archaea of group 2. Labeling experiments suggested that both the bacteria and the *Archaea* were involved in the n-damo reaction (Raghoebarsing et al., 2006). However, later studies revealed that the n-damo reaction could be performed by the single bacterial species (Ettwig et al., 2008, 2009). Nitrite was found to be key in selecting for only the bacterial species (Hu et al., 2011). In 2010, Ettwig and colleagues assembled the complete genome of the bacterial species responsible for the n-damo process, named “*Candidatus* Methylomirabilis oxyfera” (Ettwig et al., 2010).

Several enrichment cultures of *M. oxyfera* have been obtained from different freshwater habitats (**Table 1**). *M. oxyfera* cells possess a cell envelope typical of Gram-negative bacteria with a diameter of 0.25–0.5 μm and a length of 0.8–1.1 μm (Ettwig et al., 2010; Wu et al., 2012). The measured apparent affinity constant for methane of *M. oxyfera* is <5 μM (Ettwig et al., 2008) or even <0.6 μM (Raghoebarsing et al., 2006), which is significantly lower than the affinity of sulfate-dependent AMO for methane (in the order of mM; Nauhaus et al., 2002). But it should be noted that the marine sediments used for determination of the affinity of sulfate-dependent AMO for methane described by Nauhaus et al. (2002) were not continuously shaken, which are more likely to have diffusional limitations when compared to well mixed systems described by Ettwig et al. (2008) and Raghoebarsing et al. (2006). The specific activity of *M. oxyfera* is low, 0.9–6.2 nmol ${NO}_{2}^{-}$ min^-1^ mg protein^-1^) min^-^^1^ mg protein^-^^1^ (**Table 1**). In addition, the observed growth rate of *M. oxyfera* is low, with a doubling time of 1–2 weeks (Ettwig et al., 2009).

**Table 1:** The reported enrichment cultures of *M. oxyfera*.

Inoculum	Temperature (°C)	Conversion rate	Composition (%)		Reference
		nmol ${NO}_{2}^{-}$ min^-1^ mg protein^-1^)	Bacteria	Archaea
Canal sediments	25	6.2	80	10	Raghoebarsing et al. (2006)
Canal sediments	30	3.7	70	0	Ettwig et al. (2008)
Ditch sediments	30	3.4–5.6	70	0	Ettwig et al. (2009)
Ditch sediments	30 ± 1	NR^b^	70–80	NR	Kampman et al. (2012)
Mixed inoculum^a^	22	NR	15	0	Hu et al. (2009)
Mixed inoculum	35	2.5	30	40	Hu et al. (2009)
Wastewater sludge	20–23	0.9	60–70	NR	Luesken et al. (2011a)

a Mixed inoculum including sediment from a freshwater lake, anaerobic digester sludge, and returned activated sludge from a sewage treatment plant.

b Not reported.

## MOLECULAR MECHANISMS OF INTRA-AEROBIC METABOLISM IN * M. OXYFERA*

The assembly of the complete genome of *M. oxyfera*, together with proteomic and transcriptomic analysis, allowed the prediction of the central pathways involved in methane and nitrite processing in this bacterium (Ettwig et al., 2010). *M. oxyfera* encodes, transcribes, and expresses the full repertoire of genes in the aerobic methane oxidation pathway. Conversely, *M. oxyfera* lacks some genes necessary for complete denitrification. The genes encoding the enzymes for reduction of nitrous oxide (N_2_O) to dinitrogen gas (N_2_) are missing. It is hypothesized that *M. oxyfera* is capable of producing oxygen (O_2_) via a new intra-aerobic pathway that involves the dismutation of nitric oxide (NO) into N_2_ and O_2_ based on the isotopic labeling experiments. The intracellular O_2_ produced is mainly used to oxidize methane via the well-described pathway of aerobic methanotrophs catalyzed by the particulate methane monooxygenase (pMMO) complex (Ettwig et al., 2010). The remaining O_2_ is used in normal respiration by terminal respiratory oxidases (Wu et al., 2011b).

Despite the fact that *M. oxyfera* has the ability to use O_2_ for methane oxidation, the addition of either 2 or 8% O_2_ was found to have an overall detrimental effect on this bacterial species (Luesken et al., 2012). These results suggest that *M. oxyfera* cannot use external O_2_ to oxidize methane and its O_2_ production and consumption is a tightly controlled process. But the applied oxygen concentration was possibly too high. The effect of trace oxygen on *M. oxyfera* is still unknown. In addition, it has yet to be shown that in a continuous culture with alternating oxic/anoxic conditions, *M. oxyfera* would adapt to or even benefit from microoxic conditions (Luesken et al., 2012).

## ULTRASTRUCTURE OF *M. OXYFERA*

*Methylomirabilis oxyfera* possesses an atypical polygonal cell shape that is distinct from other bacterial shapes described in the literature (Wu et al., 2012). The S-layer found in this bacterial species is assumed to maintain the atypical polygonal cell shape (Wu et al., 2012), as this layer is known to play a role in mechanical cell stabilization (Engelhardt, 2007). Further, the *M. oxyfera* genome contains genes encoding endoskeletal-like elements (like MreB and FtsZ) that are known to act as internal scaffolds that influence cell shape (Young, 2003; Margolin, 2009). Thus, the endoskeleton-like elements may also play a role in maintaining the unique cell shape (Wu et al., 2012).

There is one common ultrastructural feature of most methanotrophs: intracytoplasmic membranes (ICMs). The ICMs harbor the key enzyme for methane oxidation, pMMO. The arrangement of pMMO in the ICMs results in an increase in the amount of this enzyme (Nguyen et al., 1998). However, no ICM has been observed in *M. oxyfera* under the growth conditions used (Wu et al., 2012), and therefore, it remains to be determined where the pMMO enzyme is located in *M. oxyfera* cells.

## POTENTIAL ECOLOGICAL IMPORTANCE OF THE N-DAMO PROCESS IN NATURAL ECOSYSTEMS

Inland rivers and lakes, which often receive increased downward fluxes of nitrate from agricultural runoff and upward fluxes of methane generated by anaerobic decomposition (Conrad, 1996), provide a very suitable niche for *M. oxyfera*. *M. oxyfera*-like sequences have been identified in different river and lake sediments (Raghoebarsing et al., 2006; Ettwig et al., 2009; Deutzmann and Schink, 2011; Kojima et al., 2012). Deutzmann and Schink (2011) recently reported the natural activity of *M. oxyfera*-like bacteria in Lake Constance, an oligotrophic freshwater lake in Germany.

The n-damo process is predicted to occur close to oxic/anoxic interfaces with high concentrations of methane and nitrate (Thauer and Shima, 2006; Oremland, 2010). In addition to the reported freshwater lakes and rivers, such conditions also can be found in other freshwater habitats, such as natural wetlands and rice paddy fields, which provide suitable habitats for *M. oxyfera*. Smemo and Yavitt (2008) observed the occurrence of n-damo reaction in freshwater peatlands. Recently, we confirmed the presence of *M. oxyfera*-like bacteria in rice paddy fields in Southeast China (unpublished data). Both the 16S rRNA (qp1f-qp2r; Ettwig et al., 2009) and the *pmoA* (cmo182-cmo 568; Luesken et al., 2011b) molecular biomarkers confirmed the presence of NC10 phylum bacteria closely related to *M. oxyfera*. The detected NC10 phylum bacteria showed 96–97% 16S rRNA and 85–89% *pmoA* gene sequence identities to *M. oxyfera*, respectively. These findings indicate that the n-damo process occurs in different natural freshwater habitats where it may potentially make an important contribution to the biogeochemical cycling of carbon and nitrogen.

In addition to CO_2_, methane is also a very important greenhouse gas and is over 20-fold more effective in heat-trapping in the atmosphere than CO_2_ on a per-molecule basis (IPCC, 2001). Freshwater habitats such as natural wetlands and rice paddy fields are identified as one of the main sources (38%) of atmospheric methane (IPCC, 2001). The occurrence of the n-damo process in natural freshwater ecosystems suggests that this process may potentially play an important role in reducing methane emissions. N_2_O is another important greenhouse gas that is over 310-fold more effective in heat-trapping than CO_2_ on a per-molecule basis and is responsible for 4–5% of global warming (IPCC, 2001). N_2_O is released by denitrification reactions, but there is only a small percentage of ${NO}_{2}^{-}$ min^-1^ mg protein^-1^) converted into N_2_O in the n-damo process (Ettwig et al., 2010), further bolstering interest in the great potential of this process to reduce global warming.

With the ubiquitous use of fertilizers in agriculture, large quantities of inorganic nitrogen are discharged into riverine systems, an important cause of eutrophication of water bodies. The recovery of *M. oxyfera*-like sequences from different freshwater rivers and lakes indicates that the n-damo process may have the potential to contribute significantly to reducing eutrophication in freshwater ecosystems, as the nitrite (or nitrate) is reduced to N_2_ in this process.

## POTENTIAL APPLICATION OF THE N-DAMO PROCESS IN WASTEWATER TREATMENT SYSTEM

The conventional biological nitrogen removal process in wastewater treatment plants is based on nitrification/denitrification processes. These processes require a large amount of energy to provide the aerobic conditions for nitrification [Equation (1)] and may require extra organic matter (such as methanol) for denitrification [Equation (3)]. Methane, as a major end-product of anaerobic digestion, provides an inexpensive electron donor for denitrification (Islas-Lima et al., 2004; Modin et al., 2007).

Actually, a lot of wastewater treatment plants use the biogas for heat and electricity generation. However, the biogas contains certain amount of hydrogen sulfide which should be removed by chemical or biological methods. In contrast, the hydrogen sulfide in biogas may not be removed when the methane is used for denitrification (Modin et al., 2007). But the sulfide may result in toxicity to *M. oxyfera*, although the sulfide will also be oxidized to sulfate by nitrite. The n-damo process which could use methane to drive denitrification provides another attractive option for methane utilization in wastewater treatment plants. *M. oxyfera* uses methane as its electron donor, while heterotrophic denitrifiers use organic matter as their electron donor. Therefore, the n-damo process could significantly save cost savings on addition of organic compounds (100%) compared with the conventional denitrification process. Another advantage of the n-damo process is low sludge production due to the slow growth rate of *M. oxyfera*. The doubling time of *M. oxyfera* is 1–2 weeks under laboratory conditions (Ettwig et al., 2010). In contrast, the doubling time of most heterotrophic denitrifiers ranges from hours to days (Song et al., 2000; Tabrez Khan and Hiraishi, 2001). This indicates that the n-damo process can save at least 90% of costs for sludge treatment compared with the conventional denitrification process based on the difference in the growth rates of *M. oxyfera* and heterotrophic denitrifiers. But the n-damo process needs very good sludge retention and very good process control because regrowing sludge after a process disturbance will take a very long time. Moreover, the favorable electron acceptor for the n-damo process is nitrite (Raghoebarsing et al., 2006; Ettwig et al., 2008; Hu et al., 2011; Kampman et al., 2012). The nitrite required for n-damo process can be provided through the partial nitrification process. In the partial nitrification process, ammonium would be converted to nitrite [Equation (2)], while ammonium is converted to nitrate in the conventional nitrogen removal systems. Therefore, the power consumption for ammonium oxidation can be reduced (25%) based on Equations (2) and (4), a combination of the partial nitrification process and the n-damo process which is similar to the combination of the partial nitrification process and the anammox process (Kartal et al., 2010).

Summarizing the discussion above, the combined partial nitrification and n-damo process would offer a number of economic advantages compared to conventional nitrogen removal systems, including approximate cost savings on nitrification aeration (25%), addition of organic compounds (100%) and sludge treatment (90%). In addition, it is well known that a large quantity of N_2_O is released during conventional denitrification (Itokawa et al., 2001). As far as it is currently known, there is only a small percentage of ${NO}_{2}^{-}$ min^-1^ mg protein^-1^) converted into N_2_O, and methane could be used to drive denitrification in this process (Ettwig et al., 2010). Furthermore, methane is a non-toxic compound, whereas denitrification may result in a residual methanol in effluents that causes secondary pollution. Thus, the n-damo process also provides great environmental advantages over the conventional nitrogen removal process.

$$\begin{array}{cc} Nitrification:{NH}_{4}^{+}+{2O}_{2}\to {NO}_{3}^{-}+H_{2}O+{2H}^{+} & \left(1\right)\\ Partial\,nitrification:{NH}_{4}^{+}+{1.5O}_{2}\to {NO}_{2}^{-}+H_{2}O+{2H}^{+} & \left(2\right)\\ Denitrification:\;6{NO}_{3}^{-}+{5CH}_{3}OH\to {3N}_{2}+{6OH}^{-}+{5CO}_{2}+7H_{2}O & \left(3\right)\\ N-damo:{8NO}_{2}^{-}+{3CH}_{4}+{8H}^{+}\to {3CO}_{2}+{4N}_{2}+{10H}_{2}O & \end{array}$$

Recently, Luesken et al. (2011a) detected *M. oxyfera*-like bacteria in nine out of the ten wastewater treatment plants examined. In addition, an n-damo enrichment culture (60–70%) from wastewater sludge was obtained at ambient temperature (20–23°C). These data suggested that it could be feasible to use the n-damo process for wastewater treatment at ambient temperatures. For practical application, a partial nitrification reactor is required at ambient temperatures to provide nitrite for the n-damo process. This requires a very good selection for ammonium oxidizing bacteria above nitrite oxidizing bacteria, which could be done based on oxygen and/or nitrite limitation (Hendrickx et al., 2012). The combined system (n-damo and partial nitrification) that is most likely to be feasible is a two-stage system with first the n-damo reactor, followed with a partial nitrification reactor from which nitrite is recycled to the n-damo reactor. This prevents stripping (by aeration) of methane from the liquid and it avoids a competition for methane between n-damo and aerobic methane oxidation (Kampman et al., 2012). But future research should focus on whether the trace of oxygen present in the effluent from the partial nitrification reactor could have an adverse effect on the activity of the* M. oxyfera* in the n-damo reactor.

Furthermore, Luesken et al. (2011c) and Zhu et al. (2011) obtained two cocultures of *M. oxyfera* and anaerobic ammonium-oxidizing (anammox) bacteria. The anammox process, which couples the reduction of nitrite to the oxidation of ammonium, has already been recognized as a cost-effective nitrogen removal process (Kartal et al., 2010). The coculture of *M. oxyfera* and anammox bacteria would be able to remove methane, ammonium, and nitrite simultaneously. The methane and ammonium in the coculture can be oxidized by *M. oxyfera* and anammox bacteria, respectively, both using nitrite as electron acceptor. Ammonium and methane often co-exist in effluent from an anaerobic system because they are major end products of anaerobic digestion. The required nitrite for the anammox process and the n-damo process could be provided by partial nitrification process. Therefore, a coculture of *M. oxyfera* and anammox bacteria provides another way to remove nitrogen from wastewater. A total nitrogen removal rate of 0.1 kg N m^-^^3^ d^-^^1^ was obtained from one reported cocultures at 30°C (Luesken et al., 2011c). In addition, Zhu et al. (2011) reported a total nitrogen removal rate of 0.2 kg N m^-^^3^ d^-^^1^ in the coculture of *M. oxyfera* and anammox bacteria at room temperature. This demonstrates that the application of such a coculture for wastewater nitrogen removal may be feasible in the near future.

## PERSPECTIVE

The n-damo process is one of the latest discoveries linking the carbon and nitrogen cycles. So far, while several interesting features of *M. oxyfera* have been discovered, the detailed physiological and biochemical properties of this bacterium remain unclear because of the limited availability of enrichment cultures. The acquisition of a greater number of enrichment cultures from various habitats and pure cultures of *M. oxyfera* would be helpful in unraveling the unexplored parts of this bacterium. Furthermore, the existence of the intra-aerobic pathway needs to be further examined by isolation and identification of the key enzyme(s) responsible for the conversion of NO to N_2_ and O_2_.

The occurrence and role of the n-damo process in natural ecosystems remains to be investigated further. Additional studies in different natural habitats are required to estimate the quantitative contribution of this process to reduce global warming and eutrophication. Primers based on 16S rRNA and functional genes (*pmoA*) are already available for the environmental detection of *M. oxyfera* (Ettwig et al., 2009; Deutzmann and Schink, 2011; Luesken et al., 2011b; Kojima et al., 2012). A candidate enzyme for the dismutation of NO in the intra-aerobic pathway is NO reductase (NOR; Ettwig et al., 2010). The gene *norZ* could be a valuable biomarker of the detection of *M. oxyfera.* These molecular biomarkers can be applied in a greater number of natural habitats to aid in the search for *M. oxyfera* in nature. It will be interesting to see if new groups of *M. oxyfera* will be discovered in new habitats.

The n-damo process has great potential for application in nitrogen removal in wastewater treatment plants because of its great economic and environmental benefits when compared with the conventional nitrogen removal processes. However, the long time required for enrichment of *M. oxyfera* might limit the engineering application of the n-damo process. The time required for establishing enrichment cultures for the n-damo reaction is 8–16 months (Raghoebarsing et al., 2006; Ettwig et al., 2009; Hu et al., 2009). The knowledge of nutrient requirements (macronutrients, micronutrients, and inorganic ions) and optimal environmental conditions (pH, temperature, and levels of oxygen) for the n-damo process could help in reducing the time required for enrichment of *M. oxyfera* (Kampman et al., 2012). In addition, selecting proper seeding sludge would also reduce the time required for *M. oxyfera* enrichment. Until now, only a very limited number of freshwater sediments and activated sludges have been used as the seeding sources for *M. oxyfera* enrichment. The n-damo process may occur in various natural habitats that could provide suitable seeding sludges. Another possibility for fast enrichment of *M. oxyfera* is to improve sludge retention or prevent washout of biomass. Different reactors, such as sequencing batch reactors and membrane reactors, which can efficiently retain biomass, could be tested to determine the most effective reactor configuration. Furthermore, the formation of granules of *M. oxyfera* cells can also efficiently retain biomass in reactors.

## Conflict of Interest Statement

The authors declare that the research was conducted in the absence of any commercial or financial relationships that could be construed as a potential conflict of interest.
